# Iatrogenic Cranial Pseudomeningocele on the Stroke Unit: A Lesser-Known Potential Complication After Decompressive Hemicraniectomy Following Malignant Middle Cerebral Artery Syndrome

**DOI:** 10.7759/cureus.81616

**Published:** 2025-04-02

**Authors:** Muhammad Saqib Hissan, Saugata Das

**Affiliations:** 1 Stroke Medicine, New Cross Hospital, Wolverhampton, GBR; 2 Stroke Medicine, The Royal Wolverhampton National Health Service (NHS) Trust, Wolverhampton, GBR

**Keywords:** cerebrospinal fluid (csf), decompressive hemicraniectomy, malignant mca, mca stroke, pseudomeningocele

## Abstract

This case report presents a rare complication of a large pseudomeningocele following decompressive hemicraniectomy in a young gentleman with malignant middle cerebral artery (MCA) syndrome after a large anterior circulation ischemic stroke. Our patient responded to the cerebrospinal fluid (CSF) drain before proceeding to an interval cranioplasty and made an excellent recovery over time and with vocational neurorehabilitation. Our report charts the patient's clinical journey, including diagnostic assessment, management, and outcome, and highlights the challenges in managing this uncommon complication in the stroke unit. It also underlines the need for multidisciplinary collaboration and early neurosurgical involvement in managing this complication to prevent unfavourable neurological sequelae.

## Introduction

Pseudomeningocele is a rare complication characterized by the extradural accumulation of cerebrospinal fluid (CSF) due to leakage from a surgical wound into the subcutaneous space.

It may be related to any injury to the dural sac or following incomplete closure of the dural sac as a post-operative complication. The prefix “pseudo” suggests that the collection is not an arachnoid-lined sac, unlike a true meningocele. The margin is usually composed of reactive fibrous tissue. It is more common in the spine, where surgical procedures such as a laminectomy can be complicated by the development of an iatrogenic pseudomeningocele. The typical cystic lesion has imaging features comparable to CSF on CT and MR imaging.

Postoperative pseudomeningoceles may be asymptomatic when the CSF collection is minimal. On the other hand, a significant collection can lead to headaches, nausea, and vomiting. If a high volume of CSF extravasates, it may lead to reduced intracranial pressure, which can characteristically induce a postural headache. There is a small risk that a pseudo-meningocele may become persistent or recurrent. This would, unfortunately, increase the risk of wound dehiscence, CSF fistula formation, worsening intracranial hypotension, meningitis, and, rarely, death [1]. Presentations, therefore, may vary depending on the size of the lesion. A fluctuating palpable mass enlarging with the Valsalva manoeuvre should raise clinical suspicion of this relatively rare complication.

While uncommon, they can occur following hemicraniectomy, especially in cases involving malignant MCA syndrome after a total anterior circulation ischaemic stroke [1,2]. This case report describes the formation of a large pseudomeningocele following decompressive hemicraniectomy in a young male with malignant MCA syndrome and outlines its diagnosis and management strategies.

## Case presentation

**Figure 1:**
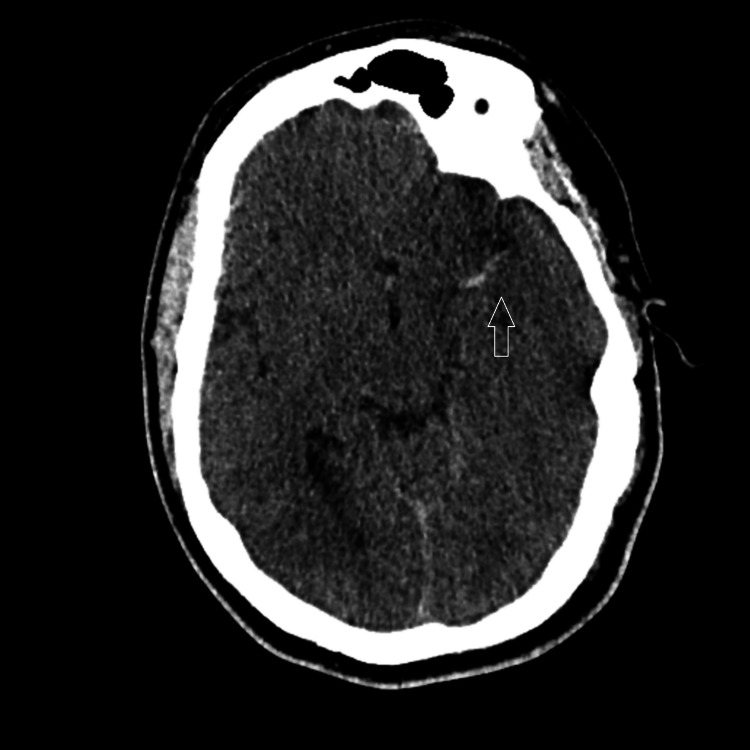
CT brain plain-axial film. Hyper-dense left MCA sign and loss of grey-white matter differentiation

A previously healthy 32-year-old male presented with a sudden onset of right-sided weakness and aphasia. He had a history of ventricular septal defect repair as a child. The initial CT brain showed a hyperdense left MCA sign (Figure 1), and computed tomography angiography (CTA) confirmed a significant occlusion in the left middle cerebral artery (MCA). Within the thrombolysis window, the patient underwent thrombolysis followed by mechanical thrombectomy at the nearest tertiary comprehensive stroke unit. Post-procedure, the patient experienced a decline in Glasgow Coma Scale (GCS) score. A repeat CT brain revealed developing malignant left MCA syndrome with massive edema and midline shift. Urgent decompressive hemicraniectomy was therefore performed.

**Figure 2:**
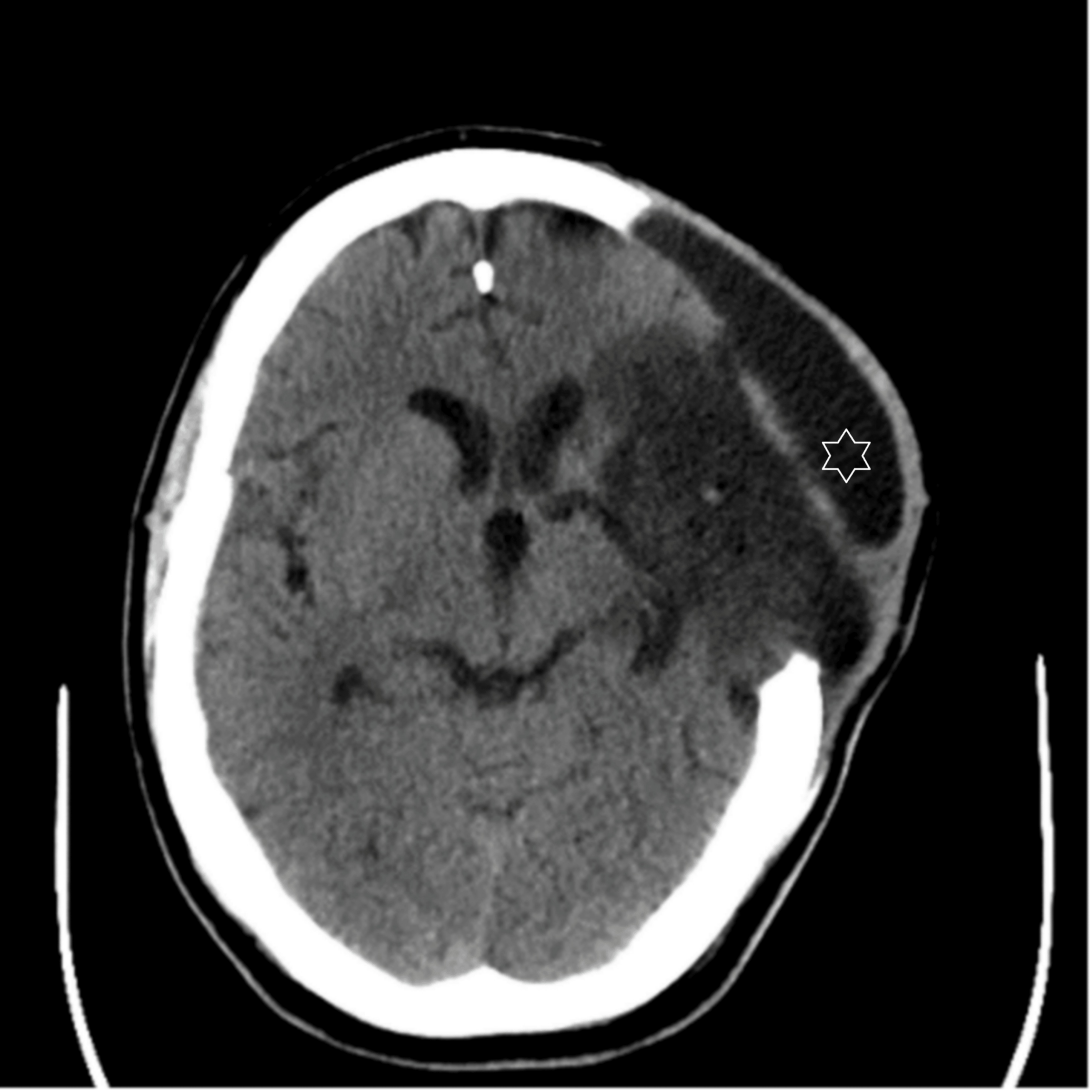
CT brain plain-axial images. Post craniectomy changes and established left MCA territory infarct. Overlying fluid density collection with a maximum thickness of 20 mm suggestive of Pseudomeningocele

Following hemicraniectomy, the patient was intubated and mechanically ventilated. He showed significant improvement, and after a stay in the intensive care unit (ICU), he was repatriated to the local stroke unit in the district general hospital. However, a few days later, the patient complained of a headache and noticed significant swelling on the ipsilateral side of the hemicraniectomy. A CT scan was performed, and this revealed an overlying fluid collection of about 20 mm through the craniectomy defect (Figure 2.) The neurosurgical team was thereby consulted.

**Figure 3:**
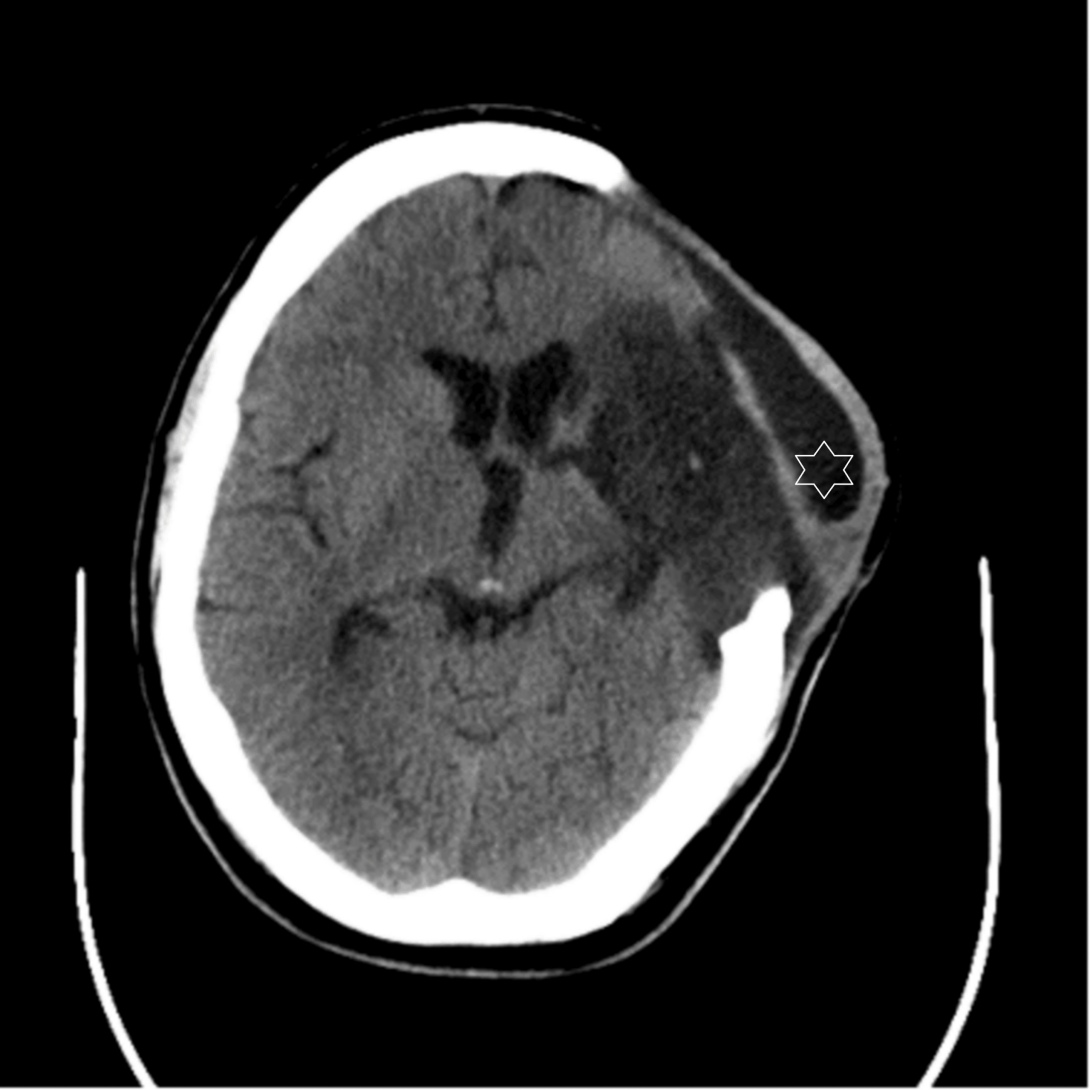
CT brain plain-axial images post lumbar puncture demonstrating reduction in swelling

A 30 mL CSF drain was advised by neurosurgery to monitor any change in the size of the swelling. A lumbar puncture was performed, draining 30 mL of CSF with an opening pressure measured at 36 cm H2O. A repeat CT scan after the procedure showed a significant reduction in swelling (Figure 3), with initial CT brain findings consistent with pseudomeningocele. He remained well thereafter with no further new symptoms and underwent extensive neurorehabilitation at the local specialist centre. He did not require further invasive surgical procedures such as a lumbar drain or external ventriculostomy drain during this time. He subsequently underwent an elective left-sided titanium cranioplasty about four months post-stroke, where he also underwent a lumbar drain procedure to prevent further CSF leakage. He underwent a further period of neurorehabilitation before returning to his home. He has since made a very good recovery and has returned to his profession as a car dealer.

## Discussion

Pseudomeningocele following hemicraniectomy is uncommon but can occur due to a persistent dural defect or inadequate closure, allowing CSF leakage into the subcutaneous space. In this case, the formation of a large pseudomeningocele was likely a sequela to the decompressive hemicraniectomy performed for the malignant transformation of MCA stroke. Hydrocephalus and subarachnoid scarring have been previously implicated as potential contributing factors [3].

Iatrogenic pseudomeningocele is not an uncommon complication following cranial and spinal surgeries, with some literature quoting an incidence rate greater than 40% [3,4]. The likely cause is a dural defect and the consequent accumulation of extradural cerebrospinal fluid (CSF) at the surgical site. However, the definite incidence after a hemicraniectomy is not clearly known due to the relative rarity of occurrence after this procedure. Not all pseudomeningoceles of the skull are symptomatic; some cause only cosmetic concerns. Potential complications; however, include wound dehiscence and infections such as meningitis, CSF fistula, and intracranial hypotension. Currently, there are no evidence-based guidelines addressing the optimal management of this condition, and treatment options include conservative management and surgical intervention based on clinical presentation [1,2,4].

Conservative management includes watchful monitoring, head elevation strategies, fluid aspiration, compressive head wrapping, or medications that decrease CSF production. Less invasive surgical options include the placement of a lumbar drain or external ventriculostomy drain, which may cause temporary CSF diversion [2,3]. Most cases resolve without sequelae. Since there is limited evidence of the most optimal initial strategy, the approach is usually individualised and dependent on local experience, expertise, and available resources. However, for persistent or recurrent pseudomeningocele, surgical intervention may be required. Open surgery for dural defect repair and subgaleal shunt placement have been noted as treatment options. In cranial cases, ventricular catheter placement, such as external ventricular drain in those developing hydrocephalus, remains a potential strategy. Intrathecal catheter placement via lumbar puncture or direct placement inside the pseudomeningocele is another option available for spinal pseudomeningocele [4,5].

Most postoperative cranial pseudomeningocele resolve spontaneously. Persistent giant cranial pseudomeningocele is extremely rare. In our case, a lumbar puncture was performed to relieve the CSF pressure and drain 30 mL of CSF, with monitoring of swelling. The patient showed a significant reduction in swelling post-procedure, supporting the diagnosis of pseudomeningocele.

## Conclusions

This case highlights the relatively rare occurrence of a large pseudomeningocele following decompressive hemicraniectomy in a young male with malignant MCA syndrome following an ischemic stroke. Our patient had timely input from the tertiary neurosurgical centre, which led to an excellent eventual outcome following vocational rehabilitation, reinforcing the need for specialist input in rare situations. Early diagnosis, appropriate monitoring, and intervention are therefore crucial in managing this uncommon complication effectively in liaison with the neurosurgical team. With advances in the field of stroke medicine and established strategies for hyperacute treatment, further research in dural closure techniques to minimise complications and optimal management of CSF extravasation are needed to better understand the risk factors, prevention, and management strategies for pseudomeningocele following hemicraniectomy in stroke patients.
